# Recent Progress on Revealing 3D Structure of Electrocatalysts Using Advanced 3D Electron Tomography: A Mini Review

**DOI:** 10.3389/fchem.2022.872117

**Published:** 2022-03-09

**Authors:** Zelin Wang, Xiaoxing Ke, Manling Sui

**Affiliations:** Beijing Key Laboratory of Microstructure and Properties of Solids, Faculty of Materials and Manufacturing, Beijing University of Technology, Beijing, China

**Keywords:** 3D electron tomography, transmission electron microscopy, electrocatalysis, nanostructures, 3D structures

## Abstract

Electrocatalysis plays a key role in clean energy innovation. In order to design more efficient, durable and selective electrocatalysts, a thorough understanding of the unique link between 3D structures and properties is essential yet challenging. Advanced 3D electron tomography offers an effective approach to reveal 3D structures by transmission electron microscopy. This mini-review summarizes recent progress on revealing 3D structures of electrocatalysts using 3D electron tomography. 3D electron tomography at nanoscale and atomic scale are discussed, respectively, where morphology, composition, porous structure, surface crystallography and atomic distribution can be revealed and correlated to the performance of electrocatalysts. (Quasi) *in-situ* 3D electron tomography is further discussed with particular focus on its impact on electrocatalysts’ durability investigation and post-treatment. Finally, perspectives on future developments of 3D electron tomography for eletrocatalysis is discussed.

## Introduction

Clean energy innovation is vital to achieving a sustainable and resilient future energy system of carbon neutralization. Electrocatalysts applied for fuel cells, water electrolyzers, and metal-air batteries, etc., offer noteworthy improvements to future energy conversion and storage technologies (Gupta, 2019; Wang, 2019; Fan et al., 2021; Tang, 2022). Thus, the development of efficient electrocatalysts for electrocatalysis, such as hydrogen evolution reaction (HER), oxygen evolution reaction (OER), oxygen reduction reaction (ORR), and CO_2_ reduction reaction, etc. has attracted much intention and investigation (Tan et al., 2018; Huang et al., 2019; Zhu, 2022).

Ongoing research on catalyst development has spent much effort on synthesizing and tuning three-dimensional (3D) nanostructures due to their excellent performance. For example, nanoparticles with concave facets, nanoframeworks with open structures, self-support porous materials derived from metal organic framework (MOF) or zeolitic imidazolate framework (ZIF), have outperformed many peer catalysts (Chen et al., 2014; Chong et al., 2018; Shen et al., 2018; Zhang et al., 2018; Cui et al., 2020; Zhang, 2020; Li et al., 2021a; Wu et al., 2021; Zhao et al., 2021). However, the characterization of nanocatalysts with complexity at all three dimensions in nanoscale or even atomic scale remains challenging yet crucial to understand the unique link between property and structure (Spivey et al., 2014; Devivaraprasad et al., 2019; Hu et al., 2019; Li et al., 2021b; Pal et al., 2021; Suter, 2021). X-ray computed tomography (XCT) and atom probe tomography (APT) have been applied to study some of the electrocatalysts in 3D (Alrwashdeh et al., 2017; He et al., 2020; Xiang et al., 2022). Nevertheless, XCT is relatively low in spatial resolution, whereas APT is less capable of resolving crystal structures. Transmission electron microscopy (TEM) is the most straightforward and widely-used characterization technique for materials, with spatial resolution spanning from sub-micron to atomic scale. 3D tomography performed in TEM is thus considered as an ideal approach to directly study the 3D structure of nanocatalysts.

**FIGURE 1:**
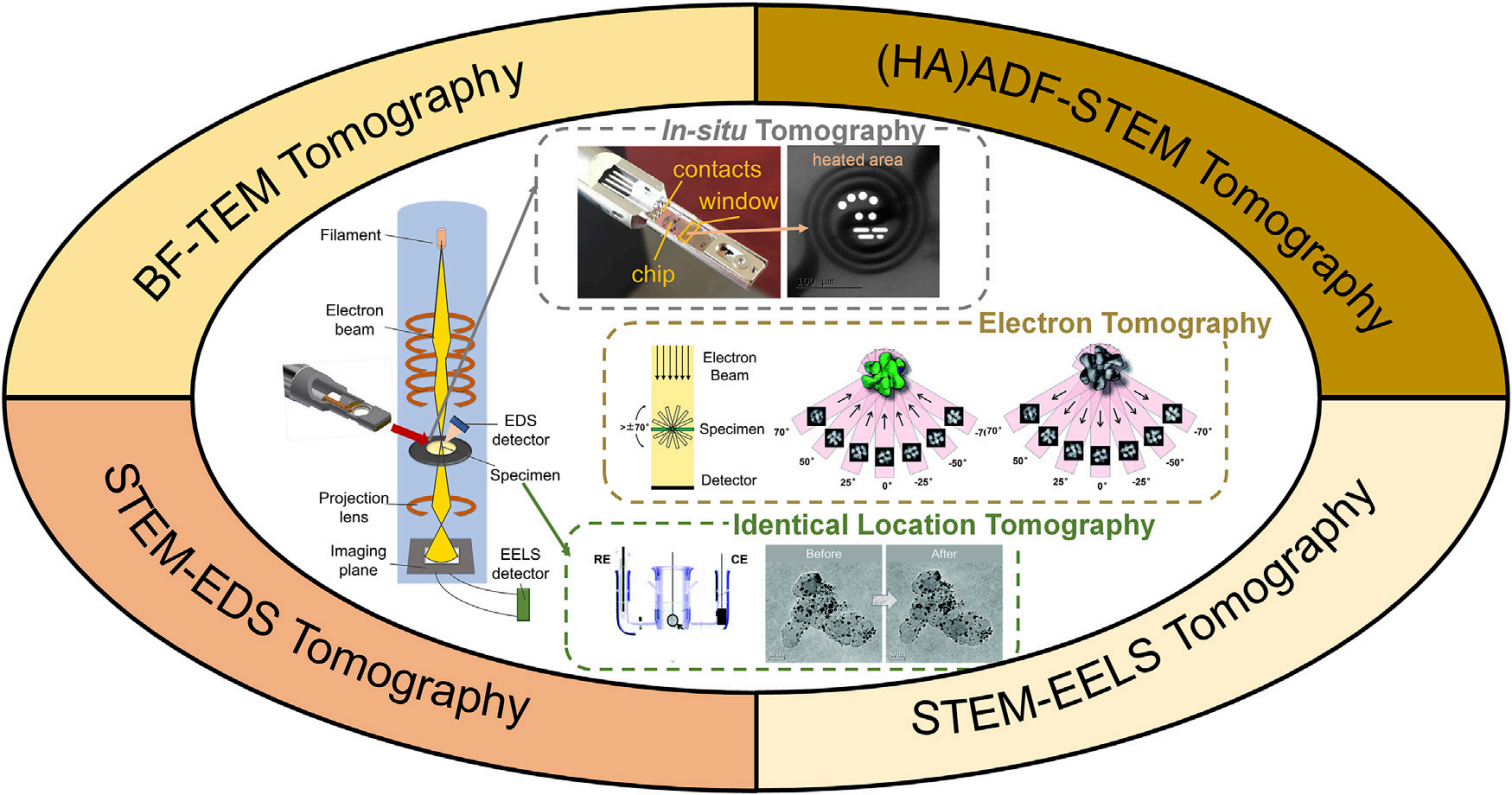
Illustration of 3D electron tomography for electrocatalysts’ characterization. WE, RE, CE are shorts for working electrode, refence electrode and counter electrode, respectively. Illustration of *in-situ* tomography was photographed from a DENSSolution *in-situ* heating holder. Illustrations of electron tomography and identical location TEM tomography were reprinted with permission from (Yu et al., 2012; Bals et al., 2014). Copyright 2014, WILEY-VCH. Copyright 2012, American Chemical Society.

Conventionally, TEM images are two-dimensional (2D) projections of 3D nano-structures, where the complexity of the unique 3D structure is partially lost. 3D electron tomography overcomes this limit by reconstructing a series of images acquired at different angles, as illustrated in Figure 1. Briefly speaking, high angle annular dark field scanning transmission electron microscopy (HAADF-STEM) images (occasionally bright-field TEM images) were taken every 1–2° across a tilt range of ±70° (Midgley and Dunin-Borkowski, 2009). Composition mapping by electron energy loss spectroscopy (EELS) and energy dispersive X-ray spectroscopy (EDX) can also be acquired for electron tomography (Haberfehlner et al., 2014; Slater et al., 2016). In such scenario, tilt increment is sometimes increased to reduce electron dose and reconstruction quality is compromised (Midgley and Dunin-Borkowski, 2009; Hungría et al., 2019). The acquired tilt series are then reconstructed using different algorithms, such as classic back projection or weighted back projection, iterative procedure, and more advanced compressive sensing, atomic electron tomography (AET) or deep-learning assisted algorithms (Midgley and Dunin-Borkowski, 2009; Zečević et al., 2013; Bals et al., 2014; Miao et al., 2016; Ding et al., 2019; Hovden and Muller, 2020; Wang, 2020). The reconstructed volume is then visualized, segmented or quantified for detailed structural investigation.

3D electron tomography has then been rapidly developed in the past decade due to the booming research in functional (nano)materials (Leary et al., 2012a; Bals et al., 2014; Ersen et al., 2015; Hovden and Muller, 2020). 3D electron tomography helps reveal the nanostructures in 3D, and thus contributes to activity and degradation study for electrocatalysis (Hungría et al., 2019; Hovden and Muller, 2020). Despite much progress has been achieved in studying catalysts and related nanomaterials by using 3D electron tomography (Zečević et al., 2013; Thomas, 2017; Zhang et al., 2017; Hungría et al., 2019), a dedicated review of electrocatalysts’ investigation by 3D electron tomography is lacking. Therefore, in this mini-review, we summarize recent applications of electron tomography towards the developments of electrocatalysts. Electrocatalysts’ morphology, composition, porous structure, surface crystallography are revealed with unprecedented details in 3D at nanoscale and atomic scale, where their correlations to electrocatalytic activity are discussed. Coupled with *in-situ* TEM and identical-location TEM, structural evolution during catalysts’ post-treatment or accelerated stress testing (AST) can be monitored in 3D, giving insights to the optimization of electrocatalysts with improved activity and durability. Finally, recent developments in 3D electron tomography and its potential applications to benefit electrocatalysts’ research are discussed.

## 3D Electron Tomography for Electrocatalysts at Nanoscale

### Distribution of Catalysts

The spatial distribution of catalysts on support materials is critical to the electrocatalytic activity. 3D electron tomography is the most straightforward approach to demonstrate the distribution in three dimensions.

**FIGURE 2:**
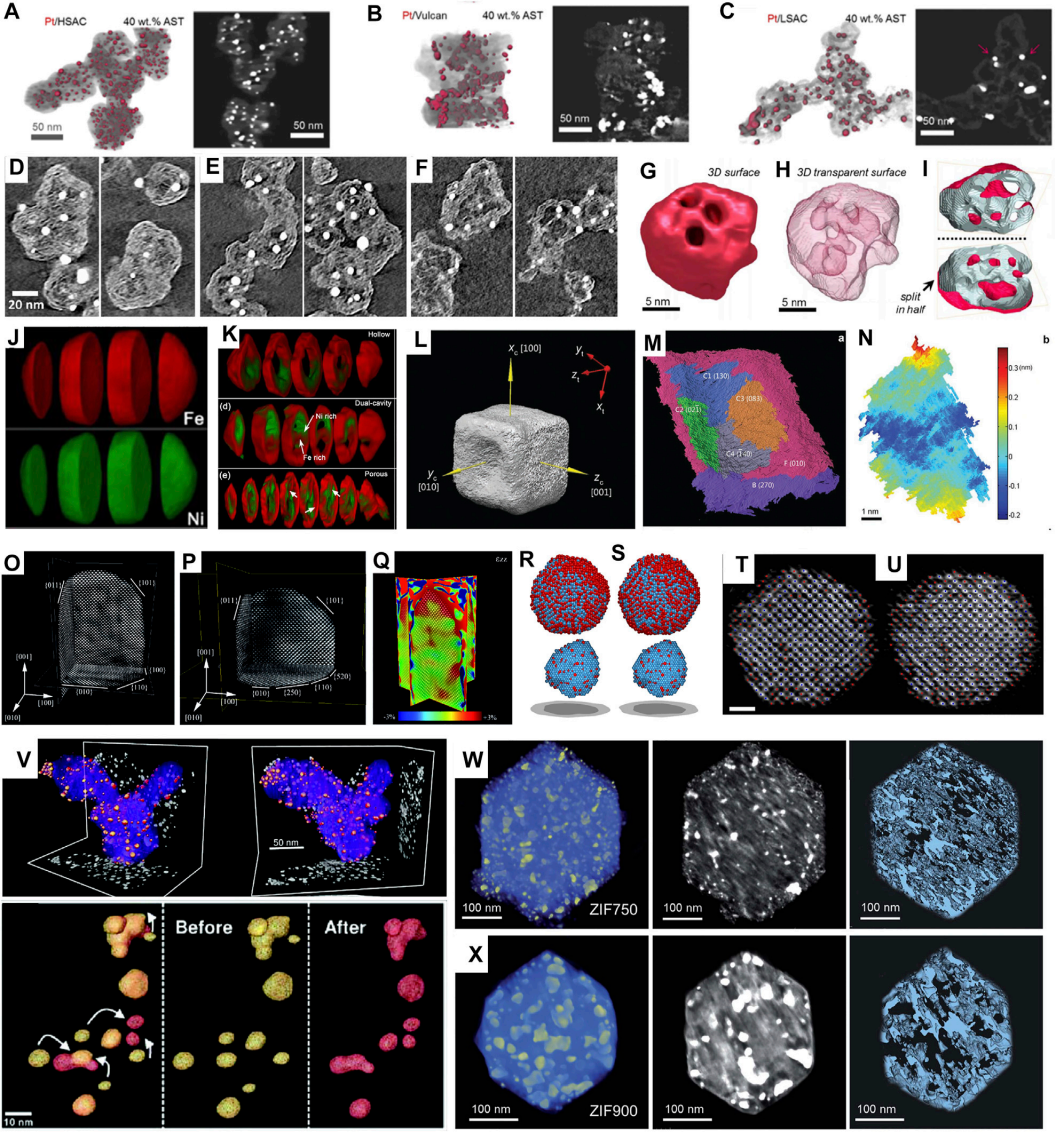
**(A–C)** 3D reconstructions and representative cross-sectional z-slices of volumes of 40 wt% Pt catalysts on **(A)** HSAC, **(B)** Vulcan, and **(C)** LSAC supports after ASTs of MEAs. Reprinted with permission from (Sneed et al., 2017). Copyright 2017, American Chemical Society. **(D–F)** Closeup of representative tomography cross sections for three different carbon blacks**,** showing the microporous structure in solid carbon (Ko et al., 2021). **(G–I)** surface render **(G)**, transparent surface render **(H)**, and surface cut in half **(I)** showing internal pore structure of electron tomography results for a Pt-Co spongy particle. (Sneed et al., 2018) **(J,K)** Chemically sensitive tomography of particles before **(J)** and after **(K)** full oxidation. Reprinted with permission from (Xia et al., 2018). Copyright 2018, American Chemical Society. **(L)** The 3D surface morphology of the Au@Pd nanocatalyst with concave facets. **(M)** The segmentation of the surface oriented along (010) of the Au@Pd concave nanocube. **(N)** Roughness map of the C3 facet in **(M)**. Reprinted with permission from (Xia et al., 2016). Copyright 2016, WILEY-VCH. **(O–Q)** atomic-resolution reconstruction of an Au nanorod. Reprinted with permission from (Bals et al., 2014). Copyright 2014, WILEY-VCH. **(R–U)** 3D atomic models (Pt in blue and Fe in red) of particle with a total annealing time of 9 min **(R,T)** and 16 min **(S,U)**, respectively, determined by AET (scalebar: 1 nm). Reprinted with permission from (Miao et al., 2020). Copyright 2020, Cambridge University Press. **(V)** 3D reconstruction of nanocatalyst particles before (yellow) and after (red) electrochemical aging. Reprinted with permission from (Yu et al., 2012). Copyright 2012, American Chemical Society **(W–X)** 3D tomographic reconstruction, corresponding orthoslices, and porous structure of ZIF annealed at **(W)** 750°C for 1 h and **(X)** 900°C for 1 h, respectively, (Wang et al., 2021b).

Carbon nanostructures are widely used as support materials for electrocatalysts, attracting most attention for 3D characterization (Banham et al., 2011; Banham et al., 2015; Ko et al., 2021). Particularly, Sneed et al. used 3D electron tomography to study the distribution of Pt on high surface area carbon (HSAC), low surface area carbon (LSAC) and commercially available Vulcan XC-72 supports (Sneed et al., 2017) (Figures 2A–C). Due to differences in pore structure and surface hydrophilicity, the ORR polarization curves showed the corresponding electrochemically active surface area (ECSA) as Pt/HSAC (69.3 m^2^/g) > Pt/Vulcan XC-72 (55.1 m^2^/g) > Pt/LSAC (46.9 m^2^/g) at same Pt loading. Very recently, Ohma et al. compared Pt loading on graphene meso-sponge (GMS) and commercially available Ketjen black (KB) (Ohma et al., 2021). The ORR activity of Pt/GMS was found to be 1.2 times higher than Pt/KB. 3D electron tomography further revealed that Pt/GMS was rich in pores to load smaller Pt particles, and hence had enhanced activity.

Other support materials have been investigated as well. Kaneko et al. showed the distribution of Pt on phthalocyanine, and found that oxygen reduction activity increased with increasing Pt dispersion (Kaneko et al., 2009). Sahin et al. revealed the distribution of Cu nanoparticles on FDU-15 used for CO_2_ reduction, and showed that nanoparticles found on the external surface of the FDU-15 were more affected by the Kirkendall effect during the oxidation (Sahin, 2018). Geerts et al. used 3D electron tomography to reveal core-shell structure of alumina supports, confirming an average distribution of ultra-small Pt nanoparticles (Geerts et al., 2021). Mariusz et al. studied how the 3D structures of Pt-loaded TiO_2_ nanotube arrays affected the formic acid electro-oxidation activity (Andrzejczuk et al., 2019). It was found by 3D electron tomography that larger nanotubes with higher porosity led to better dispersion of Pt, which was further correlated to excellent electrocatalytic performance.

### Porous Structure of Carbon Support

Carbon supports have been shown to be important in determining the ORR activity, and 3D electron tomography has been used to demonstrate the porous structure of various carbon support, including carbon nanotubes, microporous carbon and mesoporous carbon (Pei et al., 2010; Banham et al., 2011; Banham et al., 2012; Banham et al., 2015; Leppanen, 2021). Due to the low contrast of carbon in HAADF-STEM, some of the 3D electron tomography were performed using BFTEM.

The group of V. Birss studied the porous carbon structure using 3D electron tomography by BFTEM. They first studied the mesoporous colloid-imprinted carbon (CIC), and revealed the ordered porous structure with the pore size of 20–50 nm (Pei et al., 2010). Pt-loaded mesoporous CIC with smaller pore size of <15 nm and wall thickness of ∼3 nm was further studied using 3D electron tomography (Banham et al., 2011; Banham et al., 2012). They compared the structure and ORR performance with commercially available Vulcan carbon which is microporous, and demonstrated that the mesoporous carbon outperformed microporous carbon due to its capability of holding more particles inside/outside carbon support. More interestingly, they systematically investigated the 3D porous structure of a series of mesoporous CIC, and reported a correlation of ORR activity with wall thickness opposed to pore diameter (Banham et al., 2015).

More recently, Ko et al. investigated the role of porous structure in the performance of PtCo catalysts supported on “accessible” carbon (Yarlagadda et al., 2018) and conventional KB carbon (Ko et al., 2021) (Figures 2D–F). In addition to the 3D morphology of carbon structures, the pore size and dispersed Pt nanoparticle size were measured statistically. By comparing their ORR performance, it was proposed that mesoporous structure with 1–2 nm micropores and thin carbon shell was ideal, resulting in a shorter diffusion pathlength through tortuous micropores to the carbon shell and thus lower local oxygen transport resistance.

### Morphology of Loaded Nanostructures

Nanostructures’s 3D morphology, in both terms of the particle size and shape, can be better studied by 3D electron tomography. A number of electrocatalysts with different morphology have been investigated, including nanoporous and nanodendrites (Mourdikoudis et al., 2013; Geboes et al., 2016; Mourdikoudis et al., 2019; Pappert et al., 2019; Yu et al., 2019), nano platelets (Kong et al., 2013; Toth et al., 2016; González-Jiménez et al., 2018), nanoframes and nanocages (Becknell et al., 2017; Zhang et al., 2019a; Gong, 2021a), nanowires (Bu et al., 2016; Liu et al., 2020), and the most widely studied nanoparticles (Liu et al., 2012; Lu et al., 2014; Mourdikoudis et al., 2015; Londono-Calderon et al., 2017; Kang et al., 2018; Gong, 2021b; Frank et al., 2021; Leteba et al., 2021).

An interesting example was given by Kang et al., who presented programmed superstructures of AuPt nanoparticles on carbon nanotubes which showed enhanced ORR activity (Kang et al., 2018). By using 3D electron tomography, not only the octahedron shaped nanoparticles were revealed, but also the 3D assembly of nanoparticles to form a suprahelical structure of six-helix bundles along the CNT axis was demonstrated.

Concave nanoparticles have been extensively studied due to the presence of high-index facets, which can be revealed by 3D electron tomography. For example, Becknell et al. developed a Pt-Co rhombic dodecahedral nanoframe with superior ORR activity, which was about 10 and 6 times higher than that of commercial Pt/C in specific activity and mass activity, respectively, (Becknell et al., 2017). The 3D study showed that the structure resembled a highly concave particles that nearly only skeleton remained, providing high surface area. Recently, Leteba et al. reported that rhombic dodecahedral Pt-Ni nanoparticles after oleylamine aging showed enhanced ORR activity (Leteba et al., 2021). By comparing the 3D morphology of Pt-Ni nanoparticles before and after aging, concave facets with exceptionally high surface area were revealed. It was proposed that Ni leaching from surface facets produced concavity and composition of Pt_2_Ni_1_.

Similarly, comparison of 3D structures before and after electrochemical cycling proves to be helpful for catalysts’ durability study (Geboes et al., 2016; Cullen et al., 2015; Sneed et al., 2018; Ustarroz et al., 2017). For example, 3D electron tomography of Pt-Co electrocatalysts before and after AST (30 k cycles at 0.6–1.0 V) was performed by Sneed et al. (Sneed et al., 2018). It was found that relatively large-sized Pt-Co nanoparticles (>10 nm) that exhibited porous “spongy” morphology and initially had a higher Co content, were transformed into hollowed-out shells as driven by Co leaching (Figures 2G–I). Ustarroz et al. studied 3D structures of porous Pt nanoparticles before and after electrochemical cycling (Ustarroz et al., 2017). The nanostructures were found to become more compact, and the number of open pores decreased significantly.

### Composition Identification in 3D Structure

Conventional 3D electron tomography is performed using HAADF-STEM to avoid diffraction contrast. The Z-contrast HAADF-STEM can also help distinguish different compositions with large Z difference. However, for structures with similar Z numbers, HAADF-STEM is less capable and thus STEM-EDX or STEM-EELS is preferred to acquire 3D tilt series.

Han et al. studied the oxidation of Ni-Co bimetallic nanoparticles using advanced electron microscopy (Han et al., 2016). Since Ni and Co has similar Z, they can hardly be distinguished by HAADF-STEM. Therefore, Ni-Co alloyed nanoparticles were investigated by EELS-tomography. Both the porous structure and the elemental distribution in 3D were demonstrated, and segregation of Ni and Co after oxidation was clearly revealed (Figures 2J,K). Furthermore, the oxidation of Ni-Fe nanoparticles was investigated using EDX-tomography, and segregation of Ni and Fe was confirmed (Xia et al., 2018). Zhang et al. studied core-shell prussian blue analogs with Fe/Co heterogeneity and nanocage morphology for OER (Zhang et al., 2019a). By using EDX-tomography, open cage architecture with a Fe-rich shell and Co-rich core was revealed in 3D. The unique compositional and structural properties gave a high special surface area (576.2 m^2^/g) and a low overpotential (271 mV at 10 mA/cm^2^).

Recently, Gong et al. reported PdFe@Pt core-shell nanoparticle with enhanced ORR (Gong, 2021b). By using EDX tomography, they found that PdFe was uniformly dispersed in the core whereas a thin layer of Pt was covered on the surface. The same research group further studied PtCu nanoframe as ORR catalysts (Gong, 2021a). A transition from disordered PtCu to ordered intermetallic PtCu resulted in enhancement for durability, and the 3D EDX tomography revealed the nanoframe structure with even distribution of both elements in 3D.

### Exposed Surface Crystallography

It is well acknowledged that the exposed facets of nanocrystals can tune the catalytic activity due to different coordination of surface atoms. For instance, Pt (111) is more active than Pt (100) in 0.1 M HClO_4_ as for oxygen reduction reaction (Marković, 1994). Electron tomography is an effective approach to reveal catalysts’ surface facets and index exposed planes in 3D.

Take CeO_2_ for example, which is a commonly used electrocatalyst for electrocatalysis. CeO_2_ nanocrystals usually exhibit facetted morphology and are well crystallized. By reconstructing its 3D structure and correlating a few projections with corresponding HRTEM or diffraction, the orientations of nanocrystals can be resolved crystallographically. Thus, exposed facets of CeO_2_ could be understood in 3D (Kaneko et al., 2007; Tan et al., 2011). More interestingly, by using EELS -tomography, the distribution of Ce^3+^/Ce^4+^ could be reconstructed and imposed on resolved facets in 3D (Goris et al., 2014; Zhang et al., 2019b). Such manner of microstructure investigation can greatly improve the understanding of catalysts’ behavior.

2D platelets and 1D nanowires have been investigated as well (Bu et al., 2016; González-Jiménez et al., 2018; Sun et al., 2019). Sun et al. unveiled the 3D complexity of the edges of a semi-2D NiO sheets, and identified the intersecting edges between {100} and {111} nanofacets (Sun et al., 2019). Bu et al. rebuilt the 3D structure of hierarchical Pt-Co nanowires, which contained not only conventional {110} facets, but also high density of {310} facets (Bu et al., 2016). The {310} facets together with other high index facets at the surface steps or kinks were suggested as the key to the exceptional ORR activity of Pt-Co nanowires.

In addition, high-index facets could be quantified and directly related to the catalytic activity, as demonstrated on concave Au@Pd nanocubes by Xia et al. (Xia et al., 2016). By reconstructing the 3D structure of concave nanocube, the surface was further segmented to different facets and indexed (Figures 2L–N). By measuring the areas of exposed facets, the distribution of surface atoms was quantified according to their coordinate numbers. Such concave facets had 32% of the surface atoms with coordinate numbers of lower than 8, which was correlated to the catalysts’ excellent performance for ethanol oxidation.

## 3D Electron Tomography for Electrocatalysts at Atomic Scale

Since the atomic configuration at surface layers account significantly for electrocatalysis as active sites, recent developments of 3D electron tomography at atomic scale have provided unprecedented advantages in understanding the structure-property relationship at atomic scale.

Goris et al. studied Au nanorods using 3D electron tomography (Goris et al., 2012). By obtaining limited numbers of projections at a few zone axes and reconstruction based on compressive sensing, 3D structure at atomic scale and strain distribution in 3D were revealed (Figures 2O–Q). They further extended the method to Au nanoparticles, and successfully revealed the surface steps/kinks and measured lattice strain in 3D (Goris et al., 2015). More recently, Wang et al. investigated hierarchical nanoporous gold which was rich in low-coordinated sites (Wang et al., 2021a). 3D reconstruction succeeded in mapping out the coordinate environment of the nanoporous Au (particularly its surface) at single-atom level, which was correlated to its enhanced performance for CO oxidation.

Meanwhile, Miao et al. developed a reconstruction method called “atomic electron tomography (AET)” for 3D electron tomography without prior knowledge (Chen et al., 2013; Miao et al., 2016). This method was applied to a Pt-Fe nanoparticle to rebuild the 3D arrangement of atoms by Zhou et al. (Zhou et al., 2019; Miao et al., 2020). As a typical bimetallic nanocatalyst, Pt-Fe was studied at the early stage of nucleation, where the 3D reconstruction at atomic resolution revealed that the core of Pt-Fe particle was Pt-rich and remained unchanged, whereas a fraction of the surface and subsurface atoms were arranged to form L1_0_ phase during annealing (Figures 2R–U). Similarly, Pelz et al. applied AET to study the 3D atomistic structure of a multiply twinned Pd (Pelz et al., 2022). Recently, Lee et al. determined a full 3D atomic structure of a dumbbell-shaped Pt nanoparticle using deep learning assisted AET (Lee, 2022). A 3D strain tensor mapping was obtained based on 3D reconstruction, where strong tensile strain at the protruded region of the nanodumbbell was confirmed and correlated to an improved oxygen reduction reactivity on {100} facets.

## *In-Situ* and Quasi-*In-Situ* 3D Electron Tomography for Electrocatalysts’ Investigation

*In-situ* TEM is being rapidly developed in recent years. When equipped with proper holders or using dedicated environmental TEM, the microstructures’ evolution can be studied at multiple stimuli such as temperature, electric field, light, and/or in liquid/gas environment instead of vacuum. In the field of electrocatalysts’ investigation, the applications of quasi-*in-situ* electron tomography and *in-situ* electron tomography are discussed separately in this section.

### Quasi-*In-Situ* (Identical Location) 3D Electron Tomography for Degradation Study

By fixing a finder grid (e.g., gold finder grid) loaded with desired catalysts at the electrode, one can study the same catalysts before and after electrocatalysis (Hartl et al., 2011; Meier, 2012; Nikkuni et al., 2013). Developed about a decade ago, this method is called “identical location (IL)-TEM” and is particularly useful in the investigation of catalysts’ degradation mechanism (Mayrhofer et al., 2008; Arán-Ais et al., 2015; Bergmann and Roldan Cuenya, 2019). Together with electron tomography, the structural evolution during electrocatalysis can be demonstrated in 3D.

Meier et al. performed IL-tomography on the classic Pt/C catalysts under simulated start-stop conditions, where 3D structures’ changes were revealed, and degradation pathways were proposed (Meier et al., 2012). Yu et al. extended the approach to Pt-Co nanocatalysts during electrochemical aging (Yu et al., 2012). As illustrated in Figure 2V, growth of Pt shell thickness and coalescence of nanoparticles were revealed and directly correlated to loss of ECSA and activity for ORR. Pt/Ru nanocatalysts were studied using IL-tomography by Hengge et al. (Hengge et al., 2017). In addition to agglomeration and Ostwald ripening during electrocatalysis, dissolution of Ru and dealloying were revealed to account for the degradation.

### *In-Situ* 3D Electron Tomography

*In-situ* 3D electron tomography has been mainly focused on the post-treatment of electrocatalysts, such as annealing which can be simulated using *in-situ* heating in vacuum or in gas environment.

Vanrompay et al. investigated the thermal stability of Au nanostars during *in-situ* heating up to 400°C (Vanrompay et al., 2018). Local volume reductions, increments and curvature changes during annealing were unveiled in 3D. Gong et al. studied the annealing on PtCu nanoframes using *in-situ* EDX-tomography (Gong, 2021a). It was found that the transformation from disordered A1 structure to L1_1_ intermetallic structure maintained the average distribution of composition, whereas further heating led to the collapse of nanoframes and thus deteriorated ORR performance.

Recently, the pyrolysis of ZIF-67 to derive efficient ORR catalysts was investigated using *in-situ* electron tomography (Wang et al., 2021b). By simulating the annealing using *in-situ* heating up to 900°C, the microstructural evolution was monitored in 3D. They found that 750°C-annealed ZIF showed a refined hierarchical porous structure with smaller pore size and precipitated Co nanoparticles (Figures 2W–X). By correlation to *ex-situ* electrochemical measurements, a half-wave potential of 0.85 V was achieved at this pyrolysis temperature, higher than ZIF-67 annealed at other temperatures.

## Conclusion and Perspectives

In summary, we briefly review recent applications of 3D electron tomography for characterizing 3D microstructures of electrocatalysts. Finally, recent developments of 3D electron tomography and their potential applications in electrocatalysts’ characterization are discussed as below.

(1) Acquisition of tilt series. One main drawback of 3D electron tomography is long acquisition time of tilt series and therefore large electron dose. Therefore, fast tilt series was proposed and expected to be helpful for *in-situ* tomography (Vanrompay et al., 2018).
(2) STEM-EDX data processing. Although STEM-EDX has been applied to electrocatalysts as discussed in section 2.4, the STEM-EDX tilt series need special attention, particularly for quantification (Zanaga et al., 2016a; Slater et al., 2016). Alternative approaches have been developed, such as ζ-factor measurements, detectors’ shadow minimization, deep-learning-based denoising, and quantitative HAADF-STEM etc. (Zanaga et al., 2016a; Zanaga et al., 2016b; Skorikov et al., 2019; Skorikov et al., 2021).
(3) Interpretation of reconstruction. More attention needs to be paid to the interpretation of reconstruction data. Semi-quantitative methods to segment large agglomeration of particles with different size. shape and orientation were proposed (Grothausmann et al., 2011; Leary et al., 2012b). Such approaches could benefit the quantitative evaluation of catalysts in a statistical manner.
(4) Alternative methods where tilt series are not allowed. Using time series of projections, evolution of molecules could be revealed 4D (Ke et al., 2013). Cryo-STEM tomography has been performed to connect fuel cell catalyst nanostructure accessibility (Padgett et al., 2018). Electron tomography can be combined with depth-sectioning to achieve high-resolution and wide-field 3D reconstructions (Hovden et al., 2014). More recently, single 2D HAADF-STEM images of catalyst nanoparticles were demonstrated to be reconstructed in 3D by combining atom-counting approach with local minima search algorithms or molecular dynamics relaxing (van den Bos, 2018; Arslan Irmak et al., 2021; Liu et al., 2021; Albrecht et al., 2021). These approaches are expected to open up new possibilities to *in-situ* 3D characterizations such as gas-cell or liquid cell (Hodnik et al., 2016; Bergmann and Roldan Cuenya, 2019; Liu et al., 2021).

Coupled with the fast developments in microscopes and reconstruction/segmentation methods, 3D electron tomography is expected to have more impact on understanding the unique link between electrocatalysts’ structure and activity, and further paves the way to designing more efficient, selective and durable catalysts.

## References

[B1] Albrecht W. Van Aert S. Bals S. (2021). Three-Dimensional Nanoparticle Transformations Captured by an Electron Microscope. Acc. Chem. Res. 54 (5), 1189–1199. 10.1021/acs.accounts.0c00711 33566587

[B2] Alrwashdeh S. S. Manke I. Markötter H. Klages M. Göbel M. Haußmann J. (2017). In Operando Quantification of Three-Dimensional Water Distribution in Nanoporous Carbon-Based Layers in Polymer Electrolyte Membrane Fuel Cells. ACS Nano 11 (6), 5944–5949. 10.1021/acsnano.7b01720 28541662

[B3] Andrzejczuk M. Roguska A. Pisarek M. Kędzierzawski P. Lewandowska M. (2019). Effect of Pt Deposits on TiO2 Electrocatalytic Activity Highlighted by Electron Tomography. ACS Appl. Mater. Inter. 11 (20), 18841–18848. 10.1021/acsami.9b03932 31013048

[B4] Arán-Ais R. M. Yu Y. Hovden R. Solla-Gullón J. Herrero E. Feliu J. M. (2015). Identical Location Transmission Electron Microscopy Imaging of Site-Selective Pt Nanocatalysts: Electrochemical Activation and Surface Disordering. J. Am. Chem. Soc. 137 (47), 14992–14998. 10.1021/jacs.5b09553 26524187

[B5] Arslan Irmak E. Liu P. Bals S. Van Aert S. (2021). 3D Atomic Structure of Supported Metallic Nanoparticles Estimated from 2D ADF STEM Images: A Combination of Atom-Counting and a Local Minima Search Algorithm. Small Methods 5 (12), e2101150. 10.1002/smtd.202101150 34928008

[B6] Bals S. Goris B. Liz-Marzán L. M. Van Tendeloo G. (2014). Three-dimensional Characterization of noble-metal Nanoparticles and Their Assemblies by Electron Tomography. Angew. Chem. Int. Ed. 53 (40), 10600–10610. 10.1002/anie.201401059 25132322

[B7] Banham D. Feng F. Fürstenhaupt T. Pei K. Ye S. Birss V. (2011). Effect of Pt-Loaded Carbon Support Nanostructure on Oxygen Reduction Catalysis. J. Power Sourc. 196 (13), 5438–5445. 10.1016/j.jpowsour.2011.02.034

[B8] Banham D. Feng F. Fürstenhaupt T. Pei K. Ye S. Birss V. (2015). Novel Mesoporous Carbon Supports for PEMFC Catalysts. Catalysts 5 (3), 1046–1067. 10.3390/catal5031046

[B9] Banham D. Feng F. Fürstenhaupt T. Ye S. Birss V. (2012). First Time Investigation of Pt Nanocatalysts Deposited inside Carbon Mesopores of Controlled Length and Diameter. J. Mater. Chem. 22 (15), 7164–7171. 10.1039/c2jm00137c

[B10] Becknell N. Son Y. Kim D. Li D. Yu Y. Niu Z. (2017). Control of Architecture in Rhombic Dodecahedral Pt-Ni Nanoframe Electrocatalysts. J. Am. Chem. Soc. 139 (34), 11678–11681. 10.1021/jacs.7b05584 28787139

[B11] Bergmann A. Roldan Cuenya B. (2019). Operando Insights into Nanoparticle Transformations during Catalysis. ACS Catal. 9 (11), 10020–10043. 10.1021/acscatal.9b01831

[B12] Bu L. Guo S. Zhang X. Shen X. Su D. Lu G. (2016). Surface Engineering of Hierarchical Platinum-Cobalt Nanowires for Efficient Electrocatalysis. Nat. Commun. 7, 11850. 10.1038/ncomms11850 27353725 PMC4931244

[B13] Chen C.-C. Zhu C. White E. R. Chiu C.-Y. Scott M. C. Regan B. C. (2013). Three-dimensional Imaging of Dislocations in a Nanoparticle at Atomic Resolution. Nature 496 (7443), 74–77. 10.1038/nature12009 23535594

[B14] Chen C. Kang Y. Huo Z. Zhu Z. Huang W. Xin H. L. (2014). Highly Crystalline Multimetallic Nanoframes with Three-Dimensional Electrocatalytic Surfaces. Science 343 (6177), 1339–1343. 10.1126/science.1249061 24578531

[B15] Chong L. Wen J. Kubal J. Sen F. G. Zou J. Greeley J. (2018). Ultralow-loading Platinum-Cobalt Fuel Cell Catalysts Derived from Imidazolate Frameworks. Science 362 (6420), 1276–1281. 10.1126/science.aau0630 30409809

[B16] Cui C. Hu X. Wen L. (2020). Recent Progress on Nanostructured Bimetallic Electrocatalysts for Water Splitting and Electroreduction of Carbon Dioxide. J. Semicond. 41 (9), 091705. 10.1088/1674-4926/41/9/091705

[B17] Cullen D. A. Lopez-Haro M. Bayle-Guillemaud P. Guetaz L. Debe M. K. Steinbach A. J. (2015). Linking Morphology with Activity through the Lifetime of Pretreated PtNi Nanostructured Thin Film Catalysts. J. Mater. Chem. A. 3 (21), 11660–11667. 10.1039/c5ta01854d

[B18] Devivaraprasad R. Nalajala N. Bera B. Neergat M. (2019). Electrocatalysis of Oxygen Reduction Reaction on Shape-Controlled Pt and Pd Nanoparticles-Importance of Surface Cleanliness and Reconstruction. Front. Chem. 7, 648. 10.3389/fchem.2019.00648 31637231 PMC6787902

[B19] Ding G. Liu Y. Zhang R. Xin H. L. (2019). A Joint Deep Learning Model to Recover Information and Reduce Artifacts in Missing-Wedge Sinograms for Electron Tomography and beyond. Sci. Rep. 9 (1), 12803. 10.1038/s41598-019-49267-x 31488874 PMC6728317

[B20] Ersen O. Florea I. Hirlimann C. Pham-Huu C. (2015). Exploring Nanomaterials with 3D Electron Microscopy. Mater. Today 18 (7), 395–408. 10.1016/j.mattod.2015.04.004

[B21] Fan J. Chen M. Zhao Z. Zhang Z. Ye S. Xu S. (2021). Bridging the gap between Highly Active Oxygen Reduction Reaction Catalysts and Effective Catalyst Layers for Proton Exchange Membrane Fuel Cells. Nat. Energ. 6 (5), 475–486. 10.1038/s41560-021-00824-7

[B22] Frank A. Gänsler T. Hieke S. Fleischmann S. Husmann S. Presser V. (2021). Structural and Chemical Characterization of MoO2/MoS2 Triple-Hybrid Materials Using Electron Microscopy in up to Three Dimensions. Nanoscale Adv. 3 (4), 1067–1076. 10.1039/d0na00806k PMC9418330 36133289

[B23] Geboes B. Ustarroz J. Sentosun K. Vanrompay H. Hubin A. Bals S. (2016). Electrochemical Behavior of Electrodeposited Nanoporous Pt Catalysts for the Oxygen Reduction Reaction. ACS Catal. 6 (9), 5856–5864. 10.1021/acscatal.6b00668

[B24] Geerts L. Geerts-Claes H. Skorikov A. Vermeersch J. Vanbutsele G. Galvita V. (2021). Spherical Core-Shell Alumina Support Particles for Model Platinum Catalysts. Nanoscale 13 (7), 4221–4232. 10.1039/d0nr08456e 33586739

[B25] Gong M. (2021). Structure Evolution of PtCu Nanoframes from Disordered to Ordered for the Oxygen Reduction Reaction. Appl. Catal. B: Environ., 282, 119617. 10.1016/j.apcatb.2020.119617

[B26] Gong M. (2021). Surface Engineering of PdFe Ordered Intermetallics for Efficient Oxygen Reduction Electrocatalysis. Chem. Eng. J., 408, 127297. 10.1016/j.cej.2020.127297

[B27] González-Jiménez I. N. Torres-Pardo A. Rano S. Laberty-Robert C. Hernández-Garrido J. C. López-Haro M. (2018). Multicationic Sr4Mn3O10 Mesostructures: Molten Salt Synthesis, Analytical Electron Microscopy Study and Reactivity. Mater. Horiz. 5 (3), 480–485. 10.1039/c7mh00952f

[B28] Goris B. Bals S. Van den Broek W. Carbó-Argibay E. Gómez-Graña S. Liz-Marzán L. M. (2012). Atomic-scale Determination of Surface Facets in Gold Nanorods. Nat. Mater 11 (11), 930–935. 10.1038/nmat3462 23085569

[B29] Goris B. De Beenhouwer J. De Backer A. Zanaga D. Batenburg K. J. Sánchez-Iglesias A. (2015). Measuring Lattice Strain in Three Dimensions through Electron Microscopy. Nano Lett. 15 (10), 6996–7001. 10.1021/acs.nanolett.5b03008 26340328 PMC4877113

[B30] Goris B. Turner S. Bals S. Van Tendeloo G. (2014). Three-Dimensional Valency Mapping in Ceria Nanocrystals. ACS Nano 8 (10), 10878–10884. 10.1021/nn5047053 25286190

[B31] Grothausmann R. Zehl G. Manke I. Fiechter S. Bogdanoff P. Dorbandt I. (2011). Quantitative Structural Assessment of Heterogeneous Catalysts by Electron Tomography. J. Am. Chem. Soc. 133 (45), 18161–18171. 10.1021/ja2032508 21916435

[B32] Gupta S. (2019). Metal Boride‐Based Catalysts for Electrochemical Water‐Splitting: A Review. Adv. Funct. Mater. 30 (1). 10.1002/adfm.201906481

[B33] Haberfehlner G. Orthacker A. Albu M. Li J. Kothleitner G. (2014). Nanoscale Voxel Spectroscopy by Simultaneous EELS and EDS Tomography. Nanoscale 6 (23), 14563–14569. 10.1039/c4nr04553j 25349984

[B34] Han L. Meng Q. Wang D. Zhu Y. Wang J. Du X. (2016). Interrogation of Bimetallic Particle Oxidation in Three Dimensions at the Nanoscale. Nat. Commun. 7, 13335. 10.1038/ncomms13335 27928998 PMC5155145

[B35] Hartl K. Hanzlik M. Arenz M. (2011). IL-TEM Investigations on the Degradation Mechanism of Pt/C Electrocatalysts with Different Carbon Supports. Energy Environ. Sci. 4 (1), 234–238. 10.1039/c0ee00248h

[B36] He Y. Guo H. Hwang S. Yang X. He Z. Braaten J. (2020). Single Cobalt Sites Dispersed in Hierarchically Porous Nanofiber Networks for Durable and High-Power PGM-free Cathodes in Fuel Cells. Adv. Mater. 32 (46), e2003577. 10.1002/adma.202003577 33058263

[B37] Hengge K. Gänsler T. Pizzutilo E. Heinzl C. Beetz M. Mayrhofer K. J. J. (2017). Accelerated Fuel Cell Tests of Anodic Pt/Ru Catalyst via Identical Location TEM: New Aspects of Degradation Behavior. Int. J. Hydrogen Energ. 42 (40), 25359–25371. 10.1016/j.ijhydene.2017.08.108

[B38] Hodnik N. Dehm G. Mayrhofer K. J. J. (2016). Importance and Challenges of Electrochemical *In Situ* Liquid Cell Electron Microscopy for Energy Conversion Research. Acc. Chem. Res. 49 (9), 2015–2022. 10.1021/acs.accounts.6b00330 27541965

[B39] Hovden R. Ercius P. Jiang Y. Wang D. Yu Y. Abruña H. D. (2014). Breaking the Crowther Limit: Combining Depth-Sectioning and Tilt Tomography for High-Resolution, Wide-Field 3D Reconstructions. Ultramicroscopy 140, 26–31. 10.1016/j.ultramic.2014.01.013 24636875

[B40] Hovden R. Muller D. A. (2020). Electron Tomography for Functional Nanomaterials. MRS Bull. 45 (4), 298–304. 10.1557/mrs.2020.87

[B41] Hu C. Zhang L. Gong J. (2019). Recent Progress Made in the Mechanism Comprehension and Design of Electrocatalysts for Alkaline Water Splitting. Energ. Environ. Sci. 12 (9), 2620–2645. 10.1039/c9ee01202h

[B42] Huang H. Yan M. Yang C. He H. Jiang Q. Yang L. (2019). Graphene Nanoarchitectonics: Recent Advances in Graphene-Based Electrocatalysts for Hydrogen Evolution Reaction. Adv. Mater. 31 (48), e1903415. 10.1002/adma.201903415 31496036

[B43] Hungría A. B. Calvino J. J. Hernández-Garrido J. C. (2019). HAADF-STEM Electron Tomography in Catalysis Research. Top. Catal. 62 (12-16), 808. 10.1007/s11244-019-01200-2

[B44] Kaneko K. Furuya K. Hungria A. B. Hernandez-Garrido J.-C. Midgley P. A. Onodera T. (2009). Nanostructural Characterization and Catalytic Analysis of Hybridized Platinum/phthalocyanine Nanocomposites. J. Electron Microsc. 58 (5), 289–294. 10.1093/jmicro/dfp027 19525368

[B45] Kaneko K. Inoke K. Freitag B. Hungria A. B. Midgley P. A. Hansen T. W. (2007). Structural and Morphological Characterization of Cerium Oxide Nanocrystals Prepared by Hydrothermal Synthesis. Nano Lett. 7 (2), 421–425. 10.1021/nl062677b 17298010

[B46] Kang E. S. Kim Y.-T. Ko Y.-S. Kim N. H. Cho G. Huh Y. H. (2018). Peptide-Programmable Nanoparticle Superstructures with Tailored Electrocatalytic Activity. Acs Nano 12 (7), 6554–6562. 10.1021/acsnano.8b01146 29842775 PMC6556112

[B47] Ke X. Turner S. Quintana M. Hadad C. Montellano-López A. Carraro M. (2013). Dynamic Motion of Ru-Polyoxometalate Ions (POMs) on Functionalized Few-Layer Graphene. Small 9 (23), 3922–3927. 10.1002/smll.201300378 23813798

[B48] Ko M. Elliot P. Venkatt Y. (2021). Revealing the Nanostructure of Mesoporous Fuel Cell Catalyst Supports for Durable, High-Power Performance. J. Electrochem. Soc. 168 (2), abe28e. 10.1149/1945-7111/abe28e

[B49] Kong D. Wang H. Cha J. J. Pasta M. Koski K. J. Yao J. (2013). Synthesis of MoS2 and MoSe2 Films with Vertically Aligned Layers. Nano Lett. 13 (3), 1341–1347. 10.1021/nl400258t 23387444

[B50] Leary R. Midgley P. A. Thomas J. M. (2012). Recent Advances in the Application of Electron Tomography to Materials Chemistry. Acc. Chem. Res. 45 (10), 1782–1791. 10.1021/ar3001102 22897395

[B51] Leary R. Saghi Z. Armbrüster M. Wowsnick G. Schlögl R. Thomas J. M. (2012). Quantitative High-Angle Annular Dark-Field Scanning Transmission Electron Microscope (HAADF-STEM) Tomography and High-Resolution Electron Microscopy of Unsupported Intermetallic GaPd2 Catalysts. J. Phys. Chem. C 116 (24), 13343–13352. 10.1021/jp212456z

[B52] Lee J. (2022). Direct Observation of Three-Dimensional Atomic Structure of Twinned Metallic Nanoparticles and Their Catalytic Properties. Nano Lett. 10.1021/acs.nanolett.1c03773 35007087

[B53] Leppanen E. (2021). Rapid Industrial Scale Synthesis of Robust Carbon Nanotube Network Electrodes for Electroanalysis. J. Electroanalytical Chem., 896, 115255. 10.1016/j.jelechem.2021.115255

[B54] Leteba G. M. Wang Y.-C. Slater T. J. A. Cai R. Byrne C. Race C. P. (2021). Oleylamine Aging of PtNi Nanoparticles Giving Enhanced Functionality for the Oxygen Reduction Reaction. Nano Lett. 21 (9), 3989–3996. 10.1021/acs.nanolett.1c00706 33899489 PMC8289299

[B55] Li C. Li P. Yang S. Zhi C. (2021). Recently Advances in Flexible Zinc Ion Batteries. J. Semicond. 42 (10), 101603. 10.1088/1674-4926/42/10/101603

[B56] Li L. Wang P. Shao Q. Huang X. (2021). Recent Progress in Advanced Electrocatalyst Design for Acidic Oxygen Evolution Reaction. Adv. Mater. 33 (50), e2004243. 10.1002/adma.202004243 33749035

[B57] Liu P. Arslan Irmak E. De Backer A. De wael A. Lobato I. Béché A. (2021). Three-dimensional Atomic Structure of Supported Au Nanoparticles at High Temperature. Nanoscale 13 (3), 1770–1776. 10.1039/d0nr08664a 33432963

[B58] Liu X. Guo R. Ni K. Xia F. Niu C. Wen B. (2020). Reconstruction-Determined Alkaline Water Electrolysis at Industrial Temperatures. Adv. Mater. 32 (40), e2001136. 10.1002/adma.202001136 32876959

[B59] Liu Z. Xin H. Yu Z. Zhu Y. Zhang J. Mundy J. A. (2012). Atomic-Scale Compositional Mapping and 3-Dimensional Electron Microscopy of Dealloyed PtCo3Catalyst Nanoparticles with Spongy Multi-Core/Shell Structures. J. Electrochem. Soc. 159 (9), F554–F559. 10.1149/2.051209jes

[B60] Londono-Calderon A. Bahena D. Jose-Yacaman M. (2017). Effects of Pt Content on the Crystallinity and Optical Properties of Ag/Pt Nanoboxes: from Solid to Single and Polycrystalline Mesoporous Nanostructures. J. Nanoparticle Res. 19 (6), 3917. 10.1007/s11051-017-3917-4

[B61] Lu N. Wang J. Xie S. Brink J. McIlwrath K. Xia Y. (2014). Aberration Corrected Electron Microscopy Study of Bimetallic Pd-Pt Nanocrystal: Core-Shell Cubic and Core-Frame Concave Structures. J. Phys. Chem. C 118 (49), 28876–28882. 10.1021/jp509849a

[B62] Marković N. M. (1994). Structural Effects in Electrocatalysis: Oxygen Reduction on Platinum Low index Single-crystal Surfaces in Perchloric Acid Solutions. J. Electroanalytical Chem. 377 (1), 249

[B63] Mayrhofer K. J. J. Meier J. C. Ashton S. J. Wiberg G. K. H. Kraus F. Hanzlik M. (2008). Fuel Cell Catalyst Degradation on the Nanoscale. Electrochemistry Commun. 10 (8), 1144–1147. 10.1016/j.elecom.2008.05.032

[B64] Meier J. C. Galeano C. Katsounaros I. Topalov A. A. Kostka A. Schüth F. (2012). Degradation Mechanisms of Pt/C Fuel Cell Catalysts under Simulated Start-Stop Conditions. ACS Catal. 2 (5), 832–843. 10.1021/cs300024h

[B65] Meier J. C. (2012). Stability Investigations of Electrocatalysts on the Nanoscale. Energ. Environ. Sci. 5 (11). 10.1039/c2ee22550f

[B66] Miao J. Ercius P. Billinge S. J. L. (2016). Atomic Electron Tomography: 3D Structures without Crystals. Science 353 (6306), aaf2157. 10.1126/science.aaf2157 27708010

[B67] Miao J. Tian X. Kim D. Zhou J. Yang Y. Yang Y. (2020). Atomic Electron Tomography: Past, Present and Future. Microsc. Microanal 26 (S2), 652–654. 10.1017/s143192762001541x

[B68] Midgley P. A. Dunin-Borkowski R. E. (2009). Electron Tomography and Holography in Materials Science. Nat. Mater 8 (4), 271–280. 10.1038/nmat2406 19308086

[B69] Mourdikoudis S. Chirea M. Altantzis T. Pastoriza-Santos I. Pérez-Juste J. Silva F. (2013). Dimethylformamide-mediated Synthesis of Water-Soluble Platinum Nanodendrites for Ethanol Oxidation Electrocatalysis. Nanoscale 5 (11), 4776–4784. 10.1039/c3nr00924f 23613112

[B70] Mourdikoudis S. Chirea M. Zanaga D. Altantzis T. Mitrakas M. Bals S. (2015). Governing the Morphology of Pt-Au Heteronanocrystals with Improved Electrocatalytic Performance. Nanoscale 7 (19), 8739–8747. 10.1039/c4nr07481e 25904481 PMC4841216

[B71] Mourdikoudis S. Montes-García V. Rodal-Cedeira S. Winckelmans N. Pérez-Juste I. Wu H. (2019). Highly Porous Palladium Nanodendrites: Wet-Chemical Synthesis, Electron Tomography and Catalytic Activity. Dalton Trans. 48 (11), 3758–3767. 10.1039/c9dt00107g 30810142

[B72] Nikkuni F. R. Ticianelli E. A. Dubau L. Chatenet M. (2013). Identical-Location Transmission Electron Microscopy Study of Pt/C and Pt-Co/C Nanostructured Electrocatalyst Aging: Effects of Morphological and Compositional Changes on the Oxygen Reduction Reaction Activity. Electrocatalysis 4 (2), 104–116. 10.1007/s12678-013-0126-5

[B73] Ohma A. (2021). Elucidation of Oxygen Reduction Reaction and Nanostructure of Platinum-Loaded Graphene Mesosponge for Polymer Electrolyte Fuel Cell Electrocatalyst. Electrochimica Acta, 370, 137705. 10.1016/j.electacta.2020.137705

[B74] Padgett E. Andrejevic N. Liu Z. Kongkanand A. Gu W. Moriyama K. (2018). Editors' Choice-Connecting Fuel Cell Catalyst Nanostructure and Accessibility Using Quantitative Cryo-STEM Tomography. J. Electrochem. Soc. 165 (3), F173–F180. 10.1149/2.0541803jes

[B75] Pal R. Poddar A. Chattaraj P. K. (2021). Atomic Clusters: Structure, Reactivity, Bonding, and Dynamics. Front. Chem. 9, 730548. 10.3389/fchem.2021.730548 34485247 PMC8415529

[B76] Pappert K. Loza K. Shviro M. Hagemann U. Heggen M. Dunin‐Borkowski R. E. (2019). Nanoscopic Porous Iridium/Iridium Dioxide Superstructures (15 Nm): Synthesis and Thermal Conversion by *In Situ* Transmission Electron Microscopy. Chem. Eur. J. 25 (47), 11048–11057. 10.1002/chem.201901623 31140211

[B77] Pei K. Banham D. Feng F. Fürstenhaupt T. Ye S. Birss V. (2010). Oxygen Reduction Activity Dependence on the Mesoporous Structure of Imprinted Carbon Supports. Electrochemistry Commun. 12 (11), 1666–1669. 10.1016/j.elecom.2010.09.023

[B78] Pelz P. M. Groschner C. Bruefach A. Satariano A. Ophus C. Scott M. C. (2022). Simultaneous Successive Twinning Captured by Atomic Electron Tomography. ACS Nano 16, 588–596. 10.1021/acsnano.1c07772 34783237

[B79] Sahin N. E. (2018). One-Pot Soft-Template Synthesis of Nanostructured Copper-Supported Mesoporous Carbon FDU-15 Electrocatalysts for Efficient CO2 Reduction. Chemphyschem 19 (11), 1371. 10.1002/cphc.201701352 29537646

[B80] Shen K. Zhang L. Chen X. Liu L. Zhang D. Han Y. (2018). Ordered Macro-Microporous Metal-Organic Framework Single Crystals. Science 359 (6372), 206–210. 10.1126/science.aao3403 29326271

[B81] Skorikov A. Albrecht W. Bladt E. Xie X. van der Hoeven J. E. S. van Blaaderen A. (2019). Quantitative 3D Characterization of Elemental Diffusion Dynamics in Individual Ag@Au Nanoparticles with Different Shapes. ACS Nano 13 (11), 13421–13429. 10.1021/acsnano.9b06848 31626527

[B82] Skorikov A. Heyvaert W. Albecht W. Pelt D. M. Bals S. (2021). Deep Learning-Based Denoising for Improved Dose Efficiency in EDX Tomography of Nanoparticles. Nanoscale 13 (28), 12242–12249. 10.1039/d1nr03232a 34241619

[B83] Slater T. J. A. Janssen A. Camargo P. H. C. Burke M. G. Zaluzec N. J. Haigh S. J. (2016). STEM-EDX Tomography of Bimetallic Nanoparticles: A Methodological Investigation. Ultramicroscopy 162, 61–73. 10.1016/j.ultramic.2015.10.007 26780684

[B84] Sneed B. T. Cullen D. A. Mukundan R. Borup R. L. More K. L. (2018). PtCo Cathode Catalyst Morphological and Compositional Changes after PEM Fuel Cell Accelerated Stress Testing. J. Electrochem. Soc. 165 (6), F3078–F3084. 10.1149/2.0091806jes

[B85] Sneed B. T. Cullen D. A. Reeves K. S. Dyck O. E. Langlois D. A. Mukundan R. (2017). 3D Analysis of Fuel Cell Electrocatalyst Degradation on Alternate Carbon Supports. ACS Appl. Mater. Inter. 9 (35), 29839–29848. 10.1021/acsami.7b09716 28809471

[B86] Spivey J. J. Krishna K. S. Kumar C. S. S. R. Dooley K. M. Flake J. C. Haber L. H. (2014). Synthesis, Characterization, and Computation of Catalysts at the Center for Atomic-Level Catalyst Design. J. Phys. Chem. C 118 (35), 20043–20069. 10.1021/jp502556u

[B87] Sun T. Wang D. Mirkin M. V. Cheng H. Zheng J. C. Richards R. M. (2019). Direct High-Resolution Mapping of Electrocatalytic Activity of Semi-two-dimensional Catalysts with Single-Edge Sensitivity. Proc. Natl. Acad. Sci. U S A. 116 (24), 11618–11623. 10.1073/pnas.1821091116 31127040 PMC6575171

[B88] Suter T. A. M. (2021). Engineering Catalyst Layers for Next‐Generation Polymer Electrolyte Fuel Cells: A Review of Design, Materials, and Methods. Adv. Energ. Mater. 11 (37), 1025. 10.1002/aenm.202101025

[B89] Tan H. Tang J. Henzie J. Li Y. Xu X. Chen T. (2018). Assembly of Hollow Carbon Nanospheres on Graphene Nanosheets and Creation of Iron-Nitrogen-Doped Porous Carbon for Oxygen Reduction. ACS Nano 12 (6), 5674–5683. 10.1021/acsnano.8b01502 29722961

[B90] Tan J. P. Y. Tan H. R. Boothroyd C. Foo Y. L. He C. B. Lin M. (2011). Three-Dimensional Structure of CeO2 Nanocrystals. J. Phys. Chem. C 115 (9), 3544–3551. 10.1021/jp1122097

[B91] Tang H. (2022). Fuel Cells with an Operational Range of –20 °C to 200 °C Enabled by Phosphoric Acid-Doped Intrinsically Ultramicroporous Membranes. Nat. Energ. 7, 153–162. 10.1038/s41560-021-00956-w

[B92] Thomas J. M. (2017). Reflections on the Value of Electron Microscopy in the Study of Heterogeneous Catalysts. Proc. R. Soc. A. 473 (2197), 20160714. 10.1098/rspa.2016.0714 28265196 PMC5312132

[B93] Toth P. S. Velický M. Bissett M. A. Slater T. J. A. Savjani N. Rabiu A. K. (2016). Asymmetric MoS2/Graphene/Metal Sandwiches: Preparation, Characterization, and Application. Adv. Mater. 28 (37), 8256–8264. 10.1002/adma.201600484 27461734

[B94] Ustarroz J. Geboes B. Vanrompay H. Sentosun K. Bals S. Breugelmans T. (2017). Electrodeposition of Highly Porous Pt Nanoparticles Studied by Quantitative 3D Electron Tomography: Influence of Growth Mechanisms and Potential Cycling on the Active Surface Area. ACS Appl. Mater. Inter. 9 (19), 16168–16177. 10.1021/acsami.7b01619 28418651

[B95] van den Bos K. H. W. (2018). Recent Breakthroughs in Scanning Transmission Electron Microscopy of Small Species. Adv. Phys. X 3 (1), 1480420. 10.1080/23746149.2018.1480420

[B96] Vanrompay H. Bladt E. Albrecht W. Béché A. Zakhozheva M. Sánchez-Iglesias A. (2018). 3D Characterization of Heat-Induced Morphological Changes of Au Nanostars by Fast *In Situ* Electron Tomography. Nanoscale 10 (48), 22792–22801. 10.1039/c8nr08376b 30512028

[B97] Wang C. (2020). 7 Å Resolution Electron Tomography Enabled by Deep‐Learning‐Aided Information Recovery. Adv. Intell. Syst. 2 (12).

[B98] Wang C. Liu H. Duan H. Li Z. Zeng P. Zou P. (2021). 3D Atomic Imaging of Low-Coordinated Active Sites in Solid-State Dealloyed Hierarchical Nanoporous Gold. J. Mater. Chem. A. 9 (45), 25513–25521. 10.1039/d1ta05942d

[B99] Wang C. (2019). Recent Progress of Metal–Air Batteries—A Mini Review. Appl. Sci. 9 (14), 787. 10.3390/app9142787

[B100] Wang Z. Ke X. Zhou K. Xu X. Jin Y. Wang H. (2021). Engineering the Structure of ZIF-Derived Catalysts by Revealing the Critical Role of Temperature for Enhanced Oxygen Reduction Reaction. J. Mater. Chem. A. 9 (34), 18515–18525. 10.1039/d1ta03036a

[B101] Wu M. Chen C. Zhao Y. Zhu E. Li Y. (2021). Atomic Regulation of PGM Electrocatalysts for the Oxygen Reduction Reaction. Front. Chem. 9, 699861. 10.3389/fchem.2021.699861 34295875 PMC8290132

[B102] Xia W. Yang Y. Meng Q. Deng Z. Gong M. Wang J. (2018). Bimetallic Nanoparticle Oxidation in Three Dimensions by Chemically Sensitive Electron Tomography and *In Situ* Transmission Electron Microscopy. ACS Nano 12 (8), 7866–7874. 10.1021/acsnano.8b02170 30080965

[B103] Xia Y. Zhong X. Ke X. Zhang G.-R. Cheng Z. Xu B.-Q. (2016). 3D Quantification of Low-Coordinate Surface Atom Density: Bridging Catalytic Activity to Concave Facets of Nanocatalysts in Fuel Cells. Small 12 (46), 6332–6337. 10.1002/smll.201601944 27670846

[B104] Xiang W. Yang N. Li X. Linnemann J. Hagemann U. Ruediger O. (2022). 3D Atomic-Scale Imaging of Mixed Co-fe Spinel Oxide Nanoparticles during Oxygen Evolution Reaction. Nat. Commun. 13 (1), 179. 10.1038/s41467-021-27788-2 35013310 PMC8748757

[B105] Yarlagadda V. Carpenter M. K. Moylan T. E. Kukreja R. S. Koestner R. Gu W. (2018). Boosting Fuel Cell Performance with Accessible Carbon Mesopores. ACS Energ. Lett. 3 (3), 618–621. 10.1021/acsenergylett.8b00186

[B106] Yu W. Batchelor-McAuley C. Wang Y.-C. Shao S. Fairclough S. M. Haigh S. J. (2019). Characterising Porosity in Platinum Nanoparticles. Nanoscale 11 (38), 17791–17799. 10.1039/c9nr06071e 31552997

[B107] Yu Y. Xin H. L. Hovden R. Wang D. Rus E. D. Mundy J. A. (2012). Three-Dimensional Tracking and Visualization of Hundreds of Pt−Co Fuel Cell Nanocatalysts during Electrochemical Aging. Nano Lett. 12 (9), 4417–4423. 10.1021/nl203920s 22201229

[B108] Zanaga D. Altantzis T. Polavarapu L. Liz-Marzán L. M. Freitag B. Bals S. (2016a). A New Method for Quantitative XEDS Tomography of Complex Heteronanostructures. Part. Part. Syst. Charact. 33 (7), 396–403. 10.1002/ppsc.201600021

[B109] Zanaga D. Altantzis T. Sanctorum J. Freitag B. Bals S. (2016b). An Alternative Approach for ζ-factor Measurement Using Pure Element Nanoparticles. Ultramicroscopy 164, 11–16. 10.1016/j.ultramic.2016.03.002 26989979

[B110] Zečević J. de Jong K. P. de Jongh P. E. (2013). Progress in Electron Tomography to Assess the 3D Nanostructure of Catalysts. Curr. Opin. Solid State. Mater. Sci. 17 (3), 115

[B111] Zhang L. Shi W. Zhang B. (2017). A Review of Electrocatalyst Characterization by Transmission Electron Microscopy. J. Energ. Chem. 26 (6), 1117–1135. 10.1016/j.jechem.2017.10.016

[B112] Zhang M. Dai Q. Zheng H. Chen M. Dai L. (2018). Novel MOF-Derived Co@N-C Bifunctional Catalysts for Highly Efficient Zn-Air Batteries and Water Splitting. Adv. Mater. 30 (10), 1705431. 10.1002/adma.201705431 29349841

[B113] Zhang S. (2020). Core-shell Motif Construction: Highly Graphitic Nitrogen-Doped Porous Carbon Electrocatalysts Using MOF-Derived Carbon@COF Heterostructures as Sacrificial Templates. Chem. Eng. J., 396. 10.1016/j.cej.2020.125154

[B114] Zhang W. Song H. Cheng Y. Liu C. Wang C. Khan M. A. N. (2019a). Core-Shell Prussian Blue Analogs with Compositional Heterogeneity and Open Cages for Oxygen Evolution Reaction. Adv. Sci. (Weinh) 6 (7), 1801901. 10.1002/advs.201801901 30989025 PMC6446613

[B115] Zhang Y. Bals S. Van Tendeloo G. (2019b). Understanding CeO2-Based Nanostructures through Advanced Electron Microscopy in 2D and 3D. Part. Part. Syst. Characterization 36 (1), 287. 10.1002/ppsc.201800287

[B116] Zhao Y. Zheng L. Jiang D. Xia W. Xu X. Yamauchi Y. (2021). Nanoengineering Metal-Organic Framework-Based Materials for Use in Electrochemical CO2 Reduction Reactions. Small 17 (16), e2006590. 10.1002/smll.202006590 33739607

[B117] Zhou J. Yang Y. Yang Y. Kim D. S. Yuan A. Tian X. (2019). Observing crystal Nucleation in Four Dimensions Using Atomic Electron Tomography. Nature 570 (7762), 500–503. 10.1038/s41586-019-1317-x 31243385

[B118] Zhu J. (2022). Gram-Scale Production of Cu3P-Cu2O Janus Nanoparticles into Nitrogen and Phosphorous Doped Porous Carbon Framework as Bifunctional Electrocatalysts for Overall Water Splitting. Chem. Eng. J. 427, 130946. 10.1016/j.cej.2021.130946

